# Climate change and health within the South African context: A thematic content analysis study of climate change and health expert interviews

**DOI:** 10.4102/phcfm.v14i1.3203

**Published:** 2022-03-30

**Authors:** Monika dos Santos, Juanette John, Rebecca Garland, Romeo Palakatsela, Arnaud Banos, Pim Martens, Bono Nemukula, Murdock Ramathuba, Faith Nkohla, Keobakile Lenyibi

**Affiliations:** 1 Department of Psychology, College of Human Sciences, University of South Africa, Pretoria, South Africa; 2 Centre National de la Recherche Scientifique (UMR IDEES), University of le Havre, Le Havre, France; 3 Smart Places, Council for Scientific and Industrial Research, Pretoria, South Africa; 4 University College Venlo, Maastricht University, Maastricht, the Netherlands; 5 National Department of Health, Pretoria, South Africa; 6 National Department of Forestry, Fisheries and the Environment, Pretoria, South Africa; 7 Deutsche Gesellschaft für Internationale Zusammenarbeit (GIZ) GmbH, South African Office, Pretoria, South Africa

**Keywords:** climate change, health, South Africa, climate change and health expert interviews, sustainable development, healthcare systems strengthening

## Abstract

**Background:**

Climate change presents an unprecedented and urgent threat to human health and survival. South Africa’s health response will require a strong and effective intersectoral organisational effort.

**Aim:**

Exploratory interview outcomes are used to advance practice and policy recommendations, as well as for broad input in the development of a draft national framework for a health risk and vulnerability assessment (RVA) for national departments.

**Setting:**

Nationally in South Africa.

**Method:**

Twenty key expert interviews were conducted with South African experts in the field of climate change and health. Interview data was analysed by means of thematic content analysis.

**Results:**

Findings suggest that previously poor communities are most at risk to the impacts of climate change on health, as well as those with underlying medical conditions. Climate change may also serve as a catalyst for improving the healthcare system overall and should serve as the conduit to do so. A draft climate change and health RVA should take into account existing frameworks and should be implemented by local government. It is also critical that the health and health system impacts from climate change are well understood, especially in light of the plans to implement the (South African) National Health Insurance (NHI) scheme.

**Conclusion:**

Practice and policy initiatives should be holistic in nature. Consideration should be given to forming a South African National Department of Climate Change, or a similar coordinating body between the various national departments in South Africa, as health intercepts with all other domains within the climate change field.

## Introduction

Climate change presents an unprecedented international and urgent health threat to sustainable development, human health and survival, thus placing human lives at risk. All health professionals have a duty to advocate for action at all levels to mitigate and adapt to climate change and can or should play a critical role in mitigating and reducing risk. However, the global health sector has been slow to recognise the impact of climate change upon health.^1^ To facilitate action to address the threat of climate change and its impact on the heath sector, the 2021 World Health Organization (WHO) Countries of Parties 26 (COP26) Special Report on Climate Change and Health provides 10 recommendations for governments to consider, namely (1) commit to a healthy recovery (post-coronavirus disease 2019 [COVID-19]); (2) our health is not negotiable; (3) harness the benefits of climate action; (4) build health resilience to climate risks; (5) create energy systems that protect and improve climate and health; (6) reimaging urban environments, transport and mobility; (7) protect and restore nature as the foundation of our health; (8) promote healthy, sustainable and resilient food systems; (9) finance a healthier, fairer and greener future to save lives and lastly, (10) listen to the health community and prescribe urgent climate action.^2^ Of these WHO COP26 recommendations, points 1, 2, 3, 4, 9 and 10 are most relevant and intercept with this research’s study objectives and findings.

Climate risk and vulnerability assessments (RVAs) have increasingly been used internationally for adaptation actions, development planning at local, national and regional levels and for the identification of climate change hotspots. The critical challenge in terms of climate change in South Africa is the way in which multiple stressors – such as the spread of human immunodeficiency virus/acquired immunodeficiency syndrome (HIV/AIDS), the effects of economic globalisation, natural disasters, the privatisation of resources and geopolitical conflict, converge with climate change.^3,4^ The need for assessments in South Africa gained significance in the context of the (South African) National Climate Change Adaptation Strategy, which was first established in 2010 as part of the United Nations Framework Convention on Climate Change (UNFCCC) Cancun Adaptation Framework. The UNFCCC Cancun Adaptation Framework has been used to complement the existing short-term national adaptation programmes of action (NAPAs).^5,6^ The strategy indicates that in order to identify high-risk locations and groups, it is important that a national vulnerability assessment of the health sector be undertaken.^5^ This project on climate change and health represents the first in South Africa that is being used to inform government practice, policy and a draft climate change and a health RVA framework.

This study forms part of a broader climate change and health scoping project commissioned by the Deutsche Gesellschaft für Internationale Zusammenarbeit (GIZ) designed to inform the South African National Department of Health (NDoH) and South African National Department of Forestry, Fisheries and the Environment (NFFE) response to climate change in the health sector. The broader scoping exercise involved a desktop scoping review (which will inform a separate article). The subject of this article includes interviews with key stakeholders in the context of climate change-related health research, intervention and policy conducted in South Africa, to inform national practice and policy development and to use as broad input in developing a draft of climate change and health RVA template and framework. The RVA framework, which is currently being refined through a successive phase of the project in consultation with district health managers and local communities, was also a project deliverable. This article only focuses on the findings of the expert interview analyses, how it relates to practice and policy recommendations and how it broadly informs the development of the draft climate change and health RVA.

## Methods

The University of South Africa (UNISA) led the scoping project, in consultation with researchers and academics from the Council for Scientific and Industrial Research (CSIR), Africa Health Placements (AHP), Centre national de la recherche scientifique (CNRS): UMR IDEES laboratory at the University of le Havre in France and the Maastricht Sustainability Institute, Maastricht University, the Netherlands. Twenty South African key expert participants in the field of climate change and public health, selected by Unisa and the CSIR, in consultation with the NDoH and the NDFFE, were interviewed. Two female climate change and health researchers from CSIR conducted 15 interviews, whilst two climate change and health Unisa researchers (one male and one female) conducted five interviews. The interviews took place between the months of July 2019 and November 2019. Where possible, interviews were conducted in person, tape-recorded and transcribed with the use of otter.ai, which is a web-based application providing speech to text transcription.^7^ Where it was not possible to interview a participant in person, interviews were conducted via Skype, audio recorded and transcribed, otter.ai was also used.

The interview data were analysed by the researchers from Unisa and the CSIR by means of thematic content analysis, with the assistance of ATLAS.ti software.^8^ In previous studies, thematic content analysis methods have been proven to be constructive in the advancement of public health interventions.^9,10^ It is implemented in the disaggregation and interpretation of core themes and is a multistep process of inquiry during interview data analysis that comprises a process of linking codes (categories and concepts) to one another via a combination of inductive and deductive reasoning.^9,10^ These categories were then coded to identify regularities. Categories that are grouped together became themes and sub-themes, which have been captured in this article by way of headings and sub-headings. Data saturation was reached when the ability to obtain additional new categories from the raw data ceased, making further coding no longer feasible. To validate interpretations, study conclusions were taken back to a subset of the participants, as well as the key stakeholders as identified by the NDoH, South Africa (in consultation with UNISA and the CSIR) during a workshop held in January 2021, for the enrichment and substantiation of analyses.^9,10^ Two international experts in climate change and health: one based at IDEES laboratory at Le Havre University, France and the other at the Maastricht Sustainability Institute of Maastricht University, the Netherlands, also provided overall quality assurance in terms of the data analysis and findings.

The interview probes were developed based on the climate change and health expertise of the Unisa and CSIR researchers. After the initial drafting of the probes, the involved national departments and the funder, as well as the two international experts, provided further commentary and refinement. Broad interview questions and probes included, but were not limited to, the following:

- Do you think that climate change can impact the health of South Africans? If no, explore why. If yes, who do you think will be most affected and in what way?
- Are there any specific geographic areas in South Africa that you think need focused attention from an intervention, research or policy perspective?
- How can climate events affect the effectiveness and efficiency of public health programmes and systems?
- What are anticipated barriers to implementing climate change and health programmes and systems?
- What could be unintended consequences to the implementation of climate change and health programmes and systems?
- Can you provide examples of climate change and health interventions?
- Can you provide examples of climate change and health research questions or issues that can help to inform interventions, practice and policy?
- What climate change and health policies are in place, or should be in place?
- How can climate change and health adaptation plans be aligned with developmental priorities? Also explore how this intersects with the planned National Health Insurance (NHI) programme.
- How can support from actors/stakeholders at multiple levels be secured? What are the challenges for doing so?
- How can climate change and health information be utilised so that it can help inform management, planning and governance?
- How can collaboration between scientists and decision-makers be improved?
- How can problems be solved in ways that fit within local knowledge frameworks?

## Results

### Participant demographics

Seven participants have a public health background, six have an environmental health background and four are in the field of both climate change and health. Six participants are from public universities, three from research councils, whilst another three participants work for the government. Table 1 indicates the industry, role and field of experience of each expert participant.

**TABLE 1:** Participant industry, role and field of experience.

Participant	Industry	Role	Field of experience
1	Local government	Government official	Environmental health
2	Environmental health institute	Policy and grassroots advocate	Environmental health, policy
3	National department	Government official	Environmental health, national policy
4	Public university	Academic	Environmental health, communicable disease control, waste management, water quality and waste water management, climate change and health
5	Provincial department	Government official	Environmental health, provincial management
6	Public university	Academic and Head of Department in the medical field	Rural health, community orientated primary care and public health
7	Private university	Managing Director in the public health field	Public health, policy, education
8	Non-governmental organisation	Managing Director in public health field	Human resources in health, telemedicine, public health
9	Research council	Researcher	Personal solar ultraviolet radiation exposure, personal dosimetry, health risk assessment and skin cancer prevention, public health
10	International public health	Consultant	Public health, policy
11	Non-governmental organisation	Advocacy	Human dimensions of global environmental change/social development
12	Public university	Academic	Socio-ecological systems, especially within the context of natural resource use and sustainable rural livelihoods in African savannas
13	Public university	Academic	Climate change, sustainable development, public health, vulnerable communities, human-environment relations, psychology
14	Weather services	Researcher	Socio-economic applications in relation to climate change
15	Mental health, international aid	Psychologist and researcher	Psychological distress associated with environmental degradation (climate change, deforestation, pollution and mining)
16	Public university	Academic and Faculty Dean in the medical field	Malaria control studies
17	Research council	Researcher	Air-borne contamination Control and biosafety facilities with specific knowledge of pharmaceutical manufacturing facilities HVAC design and validation
18	Research council	Researcher	Health infrastructure, transmission control
19	Public university	Academic and Faculty Dean in the human sciences field	Psychology, environment and health in the Global South
20	Economics-based consultancy	Consultant	Medicine with further qualifications and extensive experience in public health and health economics

HVAC, heating, ventilation, and air conditioning.

### Interview findings

#### Vulnerability and risk

**Individual attitudes:** The attitudes of individual key role players in the field were touched on by a number of participants, indicating the need for science to lead the way in climate change advocacy:

‘Only through fact-based research and science. Personal opinions, political statements, etc., should be avoided and disproved with facts gained through recognised, peer-reviewed scientific results.’ (Participant 3, government official, national department)

It was also generally felt that lay knowledge in terms of climate change exists, but not necessarily in terms of the correct terminology or context. The youth were generally considered to have more understanding with regard to climate change and relevant contexts. Social media also played a significant role in individual climate change knowledge acquisition and overall attitudes.

**Mental health and physical vulnerability:** Another key theme to arise from the data analyses is that of psychological and physical vulnerability. The impact of climate change on mental health and individual brain physiology was mentioned in particular, for example, mental health services will also need to factor in climate-change impacts on their clinical care strategies (e.g. mental health disaster preparedness interventions) and address shortage of mental health practitioners:

‘A lot of work needs to be carried out on looking at how climate change is going to influence the brain, behaviour, the emotions of people … how psychologically prepared communities are, and individuals and what we can do in the immediate aftermath of disasters. To avoid it; it’s called disaster psychology.’ (Participant 19, academic and faculty Dean, public university)

The existing health status or some inherent traits, such as albinism, may also affect people’s vulnerability; this includes both physical (which could also be related to ultraviolet ultroviolet radiation and ozone depletion, air pollution and the impact of heat) and mental health aspects, as suggested by the given quotes:

‘So, it’s not only that we look at issues of albinism, for those who are having the condition the sun is so hot, and it affects them as they go out into the sun because of the nature of their pigmentation.’ (Participant 5, government official, provincial department)

‘Those who are working at farming areas, so when the heat strikes, like climate change itself on its own, it affects your mental capacity … I’ll tell you cases of suicide – we were told that people are getting depressed in a way that they take organophosphate poisoning.’ (Participant 5, government official, provincial department)

**Geographic, household, health and community vulnerability:** Geographic areas of concern raised by participants in terms of vulnerability included specific provinces in South Africa and rural areas in particular – this included areas in Limpopo, Mpumalanga, KwaZulu-Natal and the Northern, Eastern and Western Cape.

The impact of climate change on the health of vulnerable communities was highlighted by most participants, notably on the poorest of households, those already terminally ill, lacking food security and livelihoods.

One participant referred to the impact of climate change on the air quality and how this has an impact on several diseases that people often experience. The potential change in disease occurrences because of climate change, such as the change in malaria vector patterns, heightened diarrhoeal disease and plague, was also mentioned. These disease occurrences will be intensified because of a potential lack of services. Furthermore, within the context of food security and healthcare, poverty constrains household livelihoods:

‘I think some of the people who will be most affected are people from previously disadvantaged areas, the marginalised areas, where there is already high prevalence of other diseases that can actually be augmented by changes in climate.’ (Participant 14, researcher, weather services)

The impact of climate change was found to be problematic given that most of the poor communities are lacking resources to effectively cope with the crises, and intervention was equally lacking in terms of addressing the problem. This was found to have a spiralling effect, often to the detriment of vulnerable communities. The interconnectedness of vulnerable communities, adaptation or “safety-net” strategies, including social networks, was also highlighted, as indicated by the quote here:

‘It highlights the importance of the climate impacts and climate change within a broader social and ecological context in terms of thinking about adaptation… that’s what our work pointed to as climate change certainly potentially has impacts for food security of households, but the sort of safety nets could be quite diverse be they social or environmental and of course other types of social networks would be government social grants types of safety nets.’ (Participant 12, academic, public university)

**Climate change as a risk to the health system:** The impact of climate change on vulnerable public healthcare infrastructures was also indicated as a concerning factor. All participants agreed that climate change will impact health and the health system in South Africa, particularly for those in underserviced areas, with many indicating that the impacts are already being felt:

‘If you have a clinic that doesn’t have adequate air conditioning, or even fans, and you have long waiting times and queues, it’s either going to dissuade people from accessing the health services or it’s just going to result in a greater burden in terms of fainting in the queues, demoralising staff working under really unpleasant conditions, stifling conditions … I think that climate change is also going to directly impact the actual access of the health services that are available or the effectiveness and suitability and desirability of those services.’ (Participant 12, academic, public university)

The impact of climate change in terms of supply chain issues, as well as multiple other domains such as employee wellness, food control, clinic infrastructure and medical services, was highlighted.

#### Adaptation measures, systemic approaches, ethics, research and synergism of knowledge fields

**Adaptation measures:** The interviewees recommended various adaptation strategies that should be considered in terms of climate change and health. These include clinics being fitted with cooling systems (e.g. with fans), keeping clinics open for longer and making transport available for the community to clinics. Consideration regarding implementing tax rebates and other economic incentives was also highlighted by one participant. Participants also emphasised the need to focus on mitigation and that not enough was being performed in this regard, although no mention was made of specific mitigation strategies that should be implemented. The importance of a collaborative approach to adaptation amongst national departments, as well as the need to link adaptation to local and indigenous knowledge, was also highlighted.

Participants also associated adaptation with the availability of information and data, and indicated that it should be implemented at a grassroots level. Agribusiness and its recursive effect on health and resilience were also highlighted:

‘Climate information is something that I don’t think it’s being used. Also, availability, I don’t think people know where to get the information.’ (Participant 10, consultant, international public health)

‘But I know that within the agricultural business, they do get more resilient crops and things like that, which have a knock-on effect on improving health or being more resilient within climate change context. There are always feedback loops that are important.’ (Participant 13, academic, public university)

It was also felt that meeting the (sustainable) development goals in relation to climate change will be easier for the country if health challenges are tackled.

**Systemic and synergistic approach:** Overall, participants emphasised the need for all role players to adopt a systemic approach in terms of the way in which climate change is addressed, be it from a grassroots, public health, research, education or policy-level perspective:

‘I think that there’s going to be a greater and greater need for transdisciplinary studies and work, as challenging as that can be as well, because if you put students together from all sorts of different disciplines they can easily end up doing things that they know nothing about either. But I think overall the disciplinary silos within the Western world are not good for looking holistically at interventions. I think one has to always look systemically or holistically on a global level.’ (Participant 13, academic, public university)

The need for trans- and inter-disciplinarity in research and academia, policymaking and practitioner practice was mentioned, together with the training of school and postgraduate students, as well as advocacy in general. This included government officials across spheres and levels, implementation agencies, emergency medical services, the medical community, as well as the public and students.

One participant pointed out that long-term behaviour change in the health sector was not achievable through education only – but that people did change their behaviours after a crisis (e.g. in San Francisco, during the early years of the HIV/AIDS epidemic and more recently with the COVID-19 pandemic). Participants highlighted the importance of getting climate change and health into the curriculum for doctors and nurses and in continuing education and training. However, it was observed that it is very difficult to change the curriculum. One suggestion was to allow medical students, during their public health electives, to rotate through research councils and other institutions working on climate change and health.

Highlighting climate change and health through local professional bodies via conferences and academic journals was also suggested to increase visibility and increase awareness of the issues. Private–public partnerships were highlighted as important. These can be helpful to provide funding, to increase awareness and to bring a key stakeholder (i.e. private companies) to the table.

**Climate change ethics:** One participant mentioned that industries and countries that have contributed the most to climate change (traditionally the more Western affluent countries) have failed to take ethical accountability for their actions. The importance of ethics in climate change research was also observed, and it was mentioned that ethics may also be linked to issues of trust and transparency. Sound ethics can also enhance research efficiency:

‘The ethics related to climate change. The industries who cause climate change normally do not plough much back to the affected communities. Industries in the northern hemisphere may even impact negatively on poor countries in the southern hemisphere.’ (Participant 3, government official, national department)

‘It is about addressing some of the softer side of research, which are covered most even in the ethics. That, you speak to people’s values and ensure that people feel they need to take part, because this is not only important now to you but also to them.’ (Participant 14, researcher, weather services)

**Research initiatives:** Some participants discussed current and local research programmes being undertaken in South Africa. This included malaria research, related satellite technologies and studies within the field of psychology:

‘So, I’ve got a research programme in rural Limpopo going, looking at the effect of environmental endocrine disrupting chemicals on health and specifically related to malaria.’ (Participant 16, academic and faculty Dean, public university)

‘I have a student lead project that’s doing reviews of pesticides and child psychological development … The second group of studies I’m interested in is the concept of behaviour change. And so, I’ve performed some work on behaviour change and indoor air pollution, behaviour change and lead poisoning.’ (Participant 19, academic and faculty Dean, public university)

The urgency for localised robust scientific research within the fields of climate change and health was indicated by numerous participants, with credible research being more likely to be considered by policymakers. Proposed research focal points include climate change-related: population and community displacement, water scarcity and quality, conflict, food security, spread of disease, indirect and direct impacts and the impact on mental health. One participant also mentioned the need for scientific results to be communicated in easy-to-understand language for the general public.

It was acknowledged that there are still research gaps in terms of impacts, risks and vulnerabilities. The need for strong baseline data was highlighted, as was investigating currently available data sets that are underutilised. Key components of determining the usefulness of climate information included the clarity and timing of the message, the audience and lead times. For example, it was observed that the seasonal diseases such as malaria requires, a lot of planning (such as buying chemicals, hiring seasonal workers) for those potentially impacted and needs longer lead times in order to prepare and take the necessary precautions. In addition, research outcomes and information should be used at a higher level within the government to guide planning. The need for further research on the food/energy/water nexus was also highlighted, as well as the deployability of mobile clinics and that research should be practice driven, multidisciplinary and evidence and micro-based. It was further emphasised that researchers should have a more activist or action-orientated approach.

#### Health systems policy readiness

**Actions to improve science-policy linkages:** One participant drew from their knowledge of the successes and failures of HIV/AIDS campaigns to suggest ways to improve the linkages between research and policy, thus elevating the profile of these issues in the health sector. These include the development of a Think Tank on Climate Change, perhaps selecting from experts interviewed and project team, for members. They also suggested developing a Business Case for Climate Change and Health (examples include one which is on-going at the Medical Research Council (MRC) on mental health (http://www.mrc.ac.za/intramural-research-units/HealthSystems-current-projects) and Health Economics and Epidemiology Research Office (HE^2^RO) at the University of the Witwatersrand (Wits) Health Consortium on HIV/AIDS (http://www.heroza.org/projects/cost-budget-modelling/supporting-south-african-hivtb-investment-case/).^11,12^ This will need to include health economists. In order to improve the science-policy linkages, participants highlighted the need for strong relationships and regular dialogue between researchers and policymakers. Another participant observed the difference between research that is policy relevant and policy–demand driven, and that many researchers do want their work to support policy decisions, but because of a lack of communication or poor timing, it may not have the desired impact. The need for local solutions within the context of meaningful research was emphasised.

**Intervention:** Participants emphasised that interventions should be evidence based. They highlighted that climate change and health interventions should focus on aspects such as improved infrastructure and energy generation, better utilisation of resources, employment opportunities, further research and mass means of making the general population aware of the need to protect the planet, which includes relevant education at schools and tertiary educational institutions. The importance of prevention being better than cure was emphasised by one participant (getting the basics right). It was mentioned that the NHI should explicitly incorporate interventions related to climate change.

‘It should say that climate change is something that is serious and should be addressed by the NHI. It should not be a separate thing … there should be funding and education to the municipalities, government and the national department of health to say, you will use money for climate change adaptation.’ (Participant 2, policy and grassroots advocate, environmental health institute)

The need to identify high-risk areas at a finer resolution scale was also highlighted, as well as the advancement of practice initiatives, and it was mentioned that climate change may be a catalyst for improving the healthcare system. The need for (and sometimes lack of) monitoring and tracking interventions was also raised. One participant noticed that interventions can also start small and be practical. The usefulness of cost–benefit analysis of interventions in the absence of climate change was also highlighted.

The importance of political will, good leadership and using common sense for identifying and applying interventions, as well as the need for trust and collaboration in ensuring the success of interventions, was also cited. One participant indicated that communities should be educated in terms of climate change and intervention. Testing the acceptability of interventions within communities was indicated as very important. Interventions can also have unintended consequences if not planned properly and viewed within the bigger picture.

#### Components of developing a climate change and health risk and vulnerability assessment framework

**Climate change and health risk and vulnerability assessment framework:** Participants shared their thoughts on the fields that a climate change and health RVA framework should cover. Emphasis was placed on the need to involve government and communities at a localised level and adopting a multidisciplinary approach with a group of mandated leaders to drive the process:

‘It should be multidisciplinary, I think you need the climate scientists in the room, and you need infectious disease experts or epidemiologists rather to take that kind of information and understand how disease burdens might shift. I think you need leadership in the room for the government and the private sector to understand what’s pragmatic and feasible. For building a theoretical framework or purely clinical framework, you need something that can be implemented. And I would have probably technology experts in the room.’ (Participant 8, Managing Director in public health field, non-governmental organisation)

Some participants’ expertise lay in climate change risk and vulnerability (R&V) and human dimensions of climate change and thus they provided key inputs in terms of relevant R&V methodologies and tools. They observed that R&V frameworks are often very similar.

‘[… *B*]roadly speaking, I don’t think it’s so much a case of the frameworks that are used are significantly different, which is, again … kind of makes some of the missed opportunities to align and coordinate R&V assessments across sectors … even more of a disappointment, because I think most people are taking the IPCC risk, exposure, hazards, vulnerability, [*framework*] or some derivation thereof, approach to these assessments.’ (Participant 11, advocacy, non-governmental organisation)

The missed opportunities observed here relate to the fact that a large amount of work occurs in climate change R&V across sectors and involves multiple actors, but not in a coordinated manner. Consequently, actions are not linked and thus fall through the cracks or are being duplicated. One participant observed that because R&V and adaptation are highly cross-sectoral, there must be a coordinating body at the national level to ensure that R&V assessments use similar methods and input data and can thus be combined and compared. They highlighted examples of existing spatial platforms that bring different sectoral information together, for example, (1) NDFFE’s Climate Change Information System, (2) South African R&V Atlas, (3) CSIR’s Green Book.

**Specific frameworks recommended by experts:** The first framework mentioned by the participants is a planning tool called the *City Resilience Action Planning* or *CityRAP Tool* (http://dimsur.org/tools-2/) – a set of training exercises and activities aimed at developing the capacity of local governments in sub-Saharan Africa to understand and plan actions that progressively build urban resilience and reduce urban risk^13^:

‘[…*P*]lanning tool called city rap, city, resilience, action planning, something like that. It’s basically a participatory process for cities to not only analyse their risk of vulnerability, but also focus with a resilient lens … So basically, it is a way for cities to prioritise what some of their key issues are, then to prepare roadmap to address such issues.’ (Participant 11, advocacy, non-governmental organisation)

The *Global Programme of Research on Climate Change Vulnerability, Impact and Adaptation* (PROVIA) *Guidance on Assessing Vulnerability, Impacts and Adaptation to Climate Change* (2013), developed by the United Nations Environment Programme (UNEP), is another example that was highlighted by participants. The UNEP project team was multidisciplinary and brought together social and natural scientists.^14^ The team could not decide whether to use a top-down or bottom-up approach, which participant 11 identified as ‘the main sticking point thus far in the literature on vulnerability’. Thus, in PROVIA both approaches are discussed:

‘[… *I*]n that (PROVIA) guidance, we basically had two chapters that spoke about the two different ways you could do these things. To my knowledge, there hasn’t really been a way of bringing them together entirely, effectively.’ (Particpant 11, advocacy, non-governmental organisation (NGO)).

A health vulnerability assessment tool, applicable to South African towns, is currently being developed by the South African Medical Research Council (SAMRC).^11^ It is still preliminary, but the approach to the tool and its implementation can provide input into their project. University of Pretoria’s *AitaHealth* is a tool used by community health workers. Workers visit homes and collect data, after which they do a risk assessment to identify red flags and then follow up on these.^15^

Furthermore, it is necessary to understand the distribution of resources in an R&V assessment. The use of technology such as telemedicine should be considered in areas with less access to doctors. However, this type of technology requires connection to other technologies, which are not always available in rural areas.

Overall, there was not much awareness or knowledge of existing climate and health R&Vs internationally or in South Africa, apart from those adopted by NDFFE and the WHO.

**What stakeholders should be involved in developing a health risk and vulnerability assessment framework?:** It was agreed that stakeholders should be from across disciplines and fields. The main stakeholders observed were (in no specific order): climate scientists, infectious disease experts or epidemiologists who can relate information to understanding how the disease burdens might shift, leadership, technology experts (within the context of implementing the framework), academia, community, NGOs and government (across levels and sectors). Government sectors include health, environment, water, housing and treasury; the medical community, climate adaptation experts and private companies and grass-root community members were also listed as important. In addition, one participant highlighted the need for stakeholders to coordinate these R&V fields.

**Who should implement the health risk and vulnerability assessment framework?:** The participants highlighted different aspects to be considered for how the framework should be implemented.

*The role of national government as coordinator:* It was suggested that NDFFE or the Technical Committee on Climate Change should implement the framework, as this is ‘the only way that you’re going to have any kind of coordination between what’s going on in the different sectors … they’re the common denominator’. (Participant 11, advocacy, non-governmental organisation)

*Phased implementation and flexible tool:* Participants recommended that implementation should be at a provincial, municipal and district level and one tool will not fit all. They recommended a pilot study to assess the framework before it is implemented. In addition, they suggested the framework should be applied regularly and continuously (i.e. for monitoring and evaluation purposes) and will evolve as the situation, state and questions evolve, and as more useful, detailed data are generated (such as moving towards a higher tier of assessment as per the Intergovernmental Panel on Climate Change (IPCC) methods by starting with defaults and moving towards detailed local data).

*Local government should be able to implement*: It was recommended that after the development of the climate change and health R&V tool, a local government official should be able to implement it without the need for a consultant. The tool should align questions with data from the Integrated Development Plan (IDP), so that any official would have the information at hand.

#### National Health Insurance

The participants appeared to not have a clear idea on how the NHI planning and roll-out should address climate change impacts on health. There was, however, an indication that medication, mental health and other morbidity and mortality issues would need to be factored into the NHI:

‘NHI will have to address other issues highlighted earlier, such as pharmaceutical issues, mental health problems, higher morbidity and mortality, etc.’ (Participant 3, government official, national department)

Some participants had reservations about the NHI because of the failures in the current primary health care (PHC) system:

‘But the fact that we haven’t been very successful in getting our basic PHC system, including referrals to work and it should, provided the same barriers are going to affect that. Anyway, in the health sector we just need to do the same old stuff but do it better. And then we’ll probably be quite well equipped for climate change. But as I mentioned, for example, I think there might be some unexpected things such as financing.’ (Participant 20, consultant, economics-based consultancy)

#### Collaboration and local ownership

The majority of participants observed the need for collaboration between researchers, indigenous knowledge systems, politicians and government departments. In terms of improving collaboration, one participant suggested a model that is shaped around five pillars, which include respect, joint knowledge, currency, building networks and a concerted effort towards helping the community. Whilst indigenous knowledge and frameworks were seen as important by all participants, there was some hesitancy amongst two participants as to how indigenous knowledge should be used. It was observed that many lessons can be learnt from local and indigenous knowledge systems, in dealing with climate variability in the natural environment and combining it with scientific information to improve on the information and accessibility and that this knowledge should be capitalised on more. The concern was that climate change will present problems we have never seen before; in this sense, indigenous knowledge is a start but not the end point for climate change adaptation. In addition, it was suggested that indigenous knowledge varied (i.e. one community’s indigenous knowledge is not always applicable elsewhere).

Participants also highlighted the need for local grassroots ownership in terms of climate change and health research and interventions, as well as the importance of early engagement with the community and stakeholders. There was agreement that these aspects are not always performed well during research and climate assessments. Community champions, as well as stakeholder agency, were also identified as needs.

#### Barriers

Barriers to climate change and health strategies included the capacity of the government to deal with climate change given the burdens they are already facing. One participant emphasised the need to build trust. A lack of monitoring was also seen as a barrier to the development of an effective public health programme.It was also mentioned that the medical community is not aware of or, if they are, is too overloaded with other stresses to consider the impacts of climate change on health and the health system. In addition, it was questioned whether climate change is a priority in the health sector.

The Presidential Health Summit (2018) report was mentioned as a document that highlights the priorities of the sector; however, neither climate change nor global warming is mentioned in this report at all.^16^ The Presidential Health Summit Compact (2019) only mentions climate change once under intersectoral collaborations.^17^

Furthermore, climate change was not regarded to be a priority or core function of national and provincial departments. The aspect of corruption in government departments was mentioned as a potential barrier, and the need for clear governance, transparency and accountability was highlighted – with potential oversight by international organisations.

Other barriers included clinical aspects being highlighted above environmental issues and environmental health and practitioner practice:

‘I’ve noticed one thing that within the Department of Health, that is what is valued most are clinical issues … so they see that area of environmental climate change issues as being separate from that. But I don’t blame them because this is what most of the clinicians or clinical people with clinical background have on their mind, they’ve been taught [*only*] about patient or case management and so forth …’ (Participant 5, governmental official, provincial department)

#### Governance

Participants indicate that public health funding at the local level is not prioritised. Participants also highlighted the silos that government departments tend to work in, and the resultant gaps that ensue regarding climate change because of this shortfall. One participant working in a provincial government department indicated the need to form a dedicated ‘National Department of Climate Change’, which should report directly to the president and parliament, so that there could be a consolidated approach from government’s side in dealing with climate change:

‘Climate change should not be just a portion of the responsibilities of various departments in South Africa. There should be one dedicated “Climate Change Department”, fully funded and driven by experts, to coordinate all the “loose ends” as far as climate change is concerned. Reporting should be directly to the president and to parliament.’ (Participant 3, governmental official, national department)

## Discussion

The discussion of the results focuses on the subthemes of healthcare; adaptation and mitigation; systemic approaches and innovation; research, indigenous knowledge, intervention and policy development; the NHI; vulnerability, climate change and health risk and the vulnerability framework, as well as governance and concludes with a discussion on the study’s strengths and limitations.

### Healthcare, adaptation and mitigation

Climate change may be a catalyst for improving the healthcare system overall and should serve as the conduit to do so. Holistic healthcare and climate adaptation strategies must focus on strengthening healthcare systems in terms of human resources in health, accessibility, networking and referral, infrastructure, equipment, sanitation and medication. These outcome*s* are supported in the study of Dos Santos, Howard, Kruger, Kornik and Banos (2019), which explores healthcare service sustainability within the context of climate change in the Agincourt subdistrict, Kruger to Canyons Biosphere Region in Mpumalanga, South Africa.^18^ Under-capacitated healthcare facilities in terms of human resources in health will benefit from telemedicine and cross-referral networks. However, for telemedicine to be used, the technology for such connections is necessary; this is not always the current case in rural areas. Mental health services will need to factor in climate-change impacts into their clinical care strategies.

Adaptation strategies, which have largely been neglected within the South African context, should also be considered within the context of climate change and health and healthcare structures and services. These include clinics being fitted with cooling systems, keeping clinics open for longer and making transport available for the community to travel to clinics. There needs to be a focus on mitigation as well and not only adaptation. Studies indicate that a well-functioning and resourced health system can respond deftly to stresses such as changes in disease patterns and extreme events and be able to provide the necessary services to mitigate the impact of a changing climate on health. However, a health system that is under stress, which is most evident in previously disadvantaged areas within the South African context and unable to respond to a changing climate, may exacerbate the health impacts of the people that the health system serves.^19,20^

Tax rebates and other economic incentives for pro-environmental behaviour should be considered at an individual and institutional levels. Evidence gathered on disasters in recent memory, such as the COVID-19 pandemic, can be used to catalyse interest in adaptation, planning and thinking about the future, including awareness raising and expanding people’s horizons on how this information can be used to plan.^21^

### Systemic approaches and innovation

All role players should be empowered to adopt a holistic and systemic approach through which climate change and health governance and associations are addressed, be it from a grass-root level, public health, research or policy level. These findings concur with that of Capon, Synnott and Holliday (2009) and Li, Urich and Yin (2020) who stated that systemic approaches strengthen the case for early action and the management of complicated systemic challenges.^22,23^ Trans- and inter-disciplinarily in research and academia, policymaking and practitioner practice in terms of climate change associations with health is necessitated, together with the training of school and postgraduate students (such family medicine practitioners, primary health care practitioners and other allied health professionals). The development of a Think Tank on Climate Change and Health and the development of a Business Case for Climate Change and Health should be encouraged.

### Research, indigenous knowledge, intervention and policy development

Research should be practice driven, multidisciplinary, evidence- and micro-based. Quantifying the relationship between health outcomes and climate variables is a key first step in developing an evidence-base on the impact of climate and climate change on health in South Africa. This includes synergistic feedbacks and interactions between the different impacts. There is an urgency for localised, robust scientific research, focusing on population and community displacement, water scarcity and quality, conflict, food security, spread of disease, indirect and direct impacts of climate change and health, the food/energy/water nexus, and the impact of climate change on mental health. Furthermore, interdisciplinary/transdisciplinary training of students at the postgraduate level can assist in bringing together these different disciplines. Medical students, in their public health electives, could be allowed to rotate through research councils and others working on climate change and health. These disciplines should also be addressed by the school curriculum.

Research suggests that the representation of indigenous peoples and their knowledge in climate change adaptation literature has implications for justice, as well as for the effectiveness of adaptation efforts. Indigenous knowledge attributes in terms of climate change include factual knowledge about the environment and environmental change, factual knowledge concerning the use of the environment, cultural values and worldviews and governance and social capital.^24^

Furthermore, available data sets in South Africa need to be accessible and utilised by researchers and policy makers. There is a need to identify high risk areas at a higher resolution. Climate change and health research, highlighted through local professional bodies, conferences and journals, are one way to increase the visibility of the issues and increase awareness. Professional associations should have climate change on the agenda of their meetings and conferences in a way that links to their mandate. Scientific results also need to be communicated in ways that are understood by the general public. Collaboration and co-production between researchers, indigenous knowledge systems, politicians and government departments need to be encouraged. Policymakers and researchers should be linked with research funders. Private–public partnerships can be helpful to provide funding, to increase awareness and to bring private companies, a key stakeholder, to the table. Local grassroot ownership of climate change and health research and interventions is important, and community champions should be identified. Funding research by local students may also help to bridge the gap. Research that shapes the way policy and practice in the public health sector operate, whilst being cognisant of the challenges, will result in implementable interventions using resources and expertise available at the user level.

### National Health Insurance

It is critical that the health and health system impacts from climate change are well understood, especially in light of the plans to implement the NHI scheme. The increased stress on the health sector from climate change may undermine the implementation and expansion of the NHI.^25^ Implementation of the NHI may be a catalyst for improving primary health care services and infrastructure.^26^ The NHI should be framed in a way that deals with all primary health care problems: prevention, health promotion aspect (health literacy), and should not just be curative for it to be climate resilient. Furthermore, environmental health practitioners need to be capacitated within the forthcoming NHI structure, findings of which are supported in the work of Mathee and Wright (2013).^27^ One way of structuring a health system is to start with a minimum basket of essential products that people have access to, irrespective of where they live, followed by more expensive products. Once such a system is in place, resource allocation can then be made to ensure that care is available in high-risk areas.

### Vulnerability, climate change and health risk and vulnerability assessment framework and governance

The latest IPCC definitions of climate vulnerability characterise vulnerability as a function of sensitivity and adaptive capacity (also sometimes referred to as resilience) to stresses and shocks.^28^ In combination with exposure to climate hazards and impacts, it indicates risk. Climate change may hamper poverty alleviation strategies and sustainable agriculture in South Africa as the country still relies heavily on large-scale farmers for food products.^29^ Furthermore, AIDS, which has a significant impact on structures of healthcare systems, has taken a minimum of 1 million lives each year in sub-Saharan Africa since 1998.^30^ Because of its rapid transmission and effect on the immune system, HIV/AIDS reduces the ability of the human body to recover from the shock. People living with AIDS are thus more at risk when they are exposed to climate-related hazards such as rising temperatures, water scarcity, air pollution, potential water-borne and vector-borne disease outbreaks and habitat redistributions.^30^ Risk factors for HIV transmission in southern Africa that may be aggravated by climate change include population displacement, poverty and dislocated communities, gender violence, transactional sex, commercial sex work, increased partner numbers and increased risk-taking behaviours.^28,31^ Findings of this study indicate that a coordinating body must be instituted at the national level to ensure that climate change and health RVA assessments use similar methods and input data and thus can be combined and compared. National government should coordinate the implementation of the climate change and health RVAs. The framework should be flexible and undergo a phased implementation, starting with a pilot study to assess the framework before it is implemented nationally. Once the climate change and health RVA is finalised, the local government should implement the tool. The framework should talk to other initiatives such as CSIR’s Green Book (https://greenbook.co.za) and the National Climate Change RVA Framework.^32,33^

There needs to be robust oversight, monitoring and evaluation of governmental resource utilisation, which should be undertaken by international organisations. Consideration should be given to forming a coordinating body between the various national departments. There should also be a stronger link between national and local government because provinces don’t act in terms of issues related to local municipalities. The development of a tool that can strengthen the IDP by assimilating issues and solutions from different municipalities and across sectors should also be considered.

### Study strengths and limitations

Qualitative studies are subject to more researcher bias than quantitative studies; however, transcripts and analysed texts were sent to a subset of participants and key stakeholders for their commentary on accuracy and possible enrichment. Peer review of the analyses by all authors also strengthened confidence in the interpretations yielded by the study. Also, the relatively small cohort of 20 expert participants infers representativity limitations, for example, only one community grassroots representative was included in the study. Future research agendas that make use of mixed designs with more representative samples may go a long way in improving recommendations for service delivery and policy advancement.

## Conclusion

Interview findings indicate that South Africa, as a country and people, will be particularly vulnerable to climate change and its consequential health effects, food security and overall impact on livelihoods, particularly affecting the poor. Overall, participants held the view that the predicament in access to healthcare is set to overwhelm present and prospective generations within the South African context. Responses to climate change, whether by mitigation of its effects or adaptation to them, will require strong and effective intersectoral organisation efforts within government at all levels, along with interdisciplinary research. A proposal to centralise climate change action within the national departments or to form a South African National Department of Climate Change, which reports directly to the president and parliament, has been made. As the aspect of climate change and public health intersects with virtually all other facets of government, such an initiative may go a long way to increase collaboration across borders.
